# Biological, Antifungal, and Physical Efficacy of a Denture Cleanser Formulated with *Cnidium officinale* Extracts

**DOI:** 10.3390/biomedicines12092029

**Published:** 2024-09-05

**Authors:** Myung-Jin Lee, Song-Yi Yang, Min-Kyung Kang

**Affiliations:** 1 Department of Dental Hygiene, Division of Health Science, Baekseok University, Cheonan 31065, Republic of Korea; dh.mjlee@bu.ac.kr; 2 Department of Dental Hygiene, Konyang University, Daejeon 35365, Republic of Korea; syyang@konyang.ac.kr; 3 Department of Dental Hygiene, Hanseo University, Seosan 31963, Republic of Korea

**Keywords:** antifungal efficacy, biocompatibility, *Candida albicans*, *Cnidium officinale*, oral disease, oral management

## Abstract

Background/Objectives: We aimed to assess the antifungal efficacy and impact of a denture cleanser containing *Cnidium officinale* extract on the surface characteristics of denture base materials, as well as its physical and biological properties. Methods: The experimental denture cleansers were formulated with *C. officinale* at concentrations of 100 and 150 μg/mL, combined with 1% cocamidopropyl betaine as a natural surfactant. Antifungal efficacy was evaluated using zone-of-inhibition assays against *Candida albicans*, revealing inhibition zones of 20 ± 1.8 mm for the 100 μg/mL concentration and 23.6 ± 1.6 mm for the 150 μg/mL concentration. Surface property assessments—including hardness, roughness, color stability, and solubility measurements—demonstrated no significant differences compared to the control group. Biological evaluations included the quantification of polyphenol and flavonoid content. Results: The *C. officinale*-based cleanser showed significant antifungal activity without affecting the hardness, roughness, color stability, or solubility of denture base materials. Biological tests revealed no cytotoxicity and minimal mucosal irritation. Polyphenol and flavonoid contents were quantitatively measured, revealing higher concentrations in the experimental groups, which were correlated with significant antifungal activity. These compounds are known for their roles in disrupting microbial processes and enhancing antimicrobial effects. These findings suggest that the *C. officinale*-based denture cleanser effectively inhibits *C. albicans* while preserving the physical properties of denture base materials. Conclusions: This study highlights the potential of *C. officinale* in denture cleanser formulations, promoting denture hygiene and oral health. Future research should prioritize long-term clinical evaluations and formulation optimization.

## 1. Introduction

As Republic of Korea progresses toward an aging society, there is a growing emphasis on geriatric healthcare [1], particularly concerning oral health [2]. Since 2012, there has been a gradual expansion of health insurance coverage for prosthodontic devices for older adults, reflecting a heightened concern toward their oral health [3]. Research suggests that the rise in denture usage, driven by improved insurance benefits, has highlighted the significance of prosthetic hygiene [4]. Poor denture care can lead to microbial biofilm formation, calculus deposition, halitosis, and detrimental effects on oral health [5]; therefore, ensuring rigorous denture hygiene is essential for maintaining optimal oral health [6,7].

Denture cleaning methods are typically categorized into mechanical and chemical approaches [8]. Mechanical cleaning involves using a toothbrush with denture cleansers or toothpaste [9], which can be challenging for individuals with reduced manual dexterity [7]. Chemical cleaning methods involve soaking dentures in solutions containing cleansing agents, such as powders or tablets [7]. While these chemical agents are effective at removing debris and disinfecting dentures, they can occasionally alter the denture surfaces, leading to issues such as roughness, discoloration, or unpleasant tastes [10].

A significant concern with traditional denture cleaning methods is their potential to cause irritation or toxicity to oral tissues [1]. For example, chemical agents used in denture cleansers may lead to adverse reactions, prompting an increasing demand for natural alternatives [5]. Natural denture cleansers are gaining popularity owing to their potential to minimize such adverse effects and enhance biocompatibility [5,6]. These alternatives not only align with consumer preferences for safer products but also contribute to a reduced environmental impact [7].

*Candida albicans*, the primary pathogen responsible for oral candidiasis, is also implicated in denture stomatitis [11]. Denture stomatitis, an inflammatory condition of the oral mucosa under dentures, is frequently caused by poor oral hygiene and biofilm formation [12]. Biofilms are communities of microorganisms that adhere to surfaces, making them resistant to cleaning and difficult to eliminate [13]. Effective denture cleaning is crucial in preventing denture stomatitis, which is characterized by redness, swelling, and discomfort in the denture-bearing areas [14,15].

The shift toward natural alternatives in denture care reflects a broader trend towards safer, more biocompatible products that align with consumer demand and reduce potential health risks [15,16]. Understanding these trends and the limitations of current cleaning methods underscores the need for continued research into natural solutions that can enhance denture hygiene while minimizing adverse effects on oral tissues [17]. Natural extracts offer several advantages over synthetic chemicals [18], including enhanced biocompatibility [19], lower environmental impact [20], reduced production costs, and easier accessibility [19]. They are particularly valued for their broad-spectrum bioactive properties, such as antimicrobial, antioxidant, and anti-inflammatory effects [17].

Given these advantages, there is growing interest in developing cleansers with natural substances owing to their superior biocompatibility, lower development costs, and accessibility [21]. Currently, >75% of antibacterial agents used in clinical practice are derived from or inspired by natural sources [18], indicating that natural materials may increasingly be used in oral and denture cleansers [22].

Recent research has highlighted the potent antibacterial and antioxidant properties of natural substances such as *Cnidium officinale* extracts, leading to investigations into their use in denture base resins [23]. *C. officinale*, a medicinal plant from the Umbelliferae family, is renowned for its antioxidant, antibacterial, and pharmacological properties [20,23]. Its extracts exhibit strong antimicrobial effects against a wide range of pathogens, including bacteria and fungi, and offer antioxidant and anti-inflammatory benefits [19,24].

This study is unique in its exploration of *C. officinale* as a primary ingredient in a denture cleanser, emphasizing its natural antifungal and antimicrobial properties. Unlike conventional denture cleansers, which often rely on synthetic chemicals, *C. officinale* provides a natural alternative that could minimize side effects [20]. Previous studies have demonstrated its effectiveness against *C. albicans*, a common cause of oral infections such as denture stomatitis [19,20]. With its bioactive compounds, including polyphenols and flavonoids, which contribute to antimicrobial and anti-inflammatory properties, *C. officinale* is a promising candidate for denture cleansers [24]. Its ability to maintain oral tissue health while providing antimicrobial action sets it apart from synthetic alternatives [20].

The mechanism of action of *C. officinale* involves disruption of microbial cell membranes, inhibition of microbial enzyme activity, and interference with microbial DNA replication [25]. These mechanisms collectively contribute to its ability to effectively eradicate pathogens and prevent microbial growth on denture surfaces [20]. In particular, research has shown that the antioxidant properties of *C. officinale* protect denture materials from oxidative degradation, thereby prolonging their lifespan and maintaining their structural integrity [25]. Despite these promising findings, research on their application as denture cleansers is limited.

This study aimed to evaluate the efficacy of a denture cleanser formulated with *C. officinale* extract. Specifically, it assessed the antifungal activity of the cleanser against *C. albicans* and its effects on denture base resins, including surface characteristics, physical properties, and biocompatibility. The null hypothesis posited that denture cleansers with varying concentrations of *C. officinale* extract would not (1) significantly impact the antimicrobial and biocompatibility properties or (2) alter the surface properties or dissolution of the denture material. Our objective was to determine the potential of *C. officinale* extract as an effective component in denture cleansing formulations.

## 2. Materials and Methods

### 2.1. Extraction

*C. officinale* (purity: 99.7%), sourced from the Sobaek Mountains in Gyeongsangnam-do, Republic of Korea, was commercially procured [20] and compared with a standard reference sample from the National Institute of Food and Drug Safety Evaluation. To extract bioactive compounds, 500 g of *C. officinale* leaves were finely ground and homogenized in 5 L of 70% methanol solution. The mixture was soaked at room temperature for 24 h with continuous stirring. After extraction, the mixture was filtered through Whatman filter paper #2. The filtrate was concentrated using a rotary vacuum evaporator (ETELA, Tokyo, Japan) and then freeze-dried (Ilshin Lab, Dongducheon, Republic of Korea) at −55 °C for 48 h. The resulting powder was finely pulverized with a mortar and pestle and dissolved in dimethyl sulfoxide (DMSO; Amresco, Solon, OH, USA) to prepare stock solutions of 0, 100, and 150 µg/mL.

### 2.2. Preparation of Denture Cleansers Containing C. officinale

Each *C. officinale* extract solution was incorporated into a 1% cocamidopropyl betaine solution (CocoBetaina; The Modern Co. Ltd., Bucheon, Republic of Korea), a natural surfactant, to formulate the experimental denture cleansers. This mixture was then subjected to magnetic stirring at 25 ± 1 °C for 24 h. Denture cleanser formulations were prepared as follows: the control group did not contain *C. officinale* extract. In contrast, the experimental groups were treated with *C. officinale* extract at concentrations of 100 and 150 µg/mL. A 1% natural surfactant, cocamidopropyl betaine, was incorporated into all solutions. In this study, the group with 100 µg/mL of *C. officinale* was referred to as CN 100, and the group with 150 µg/mL was referred to as CN 150. From the results of preliminary experiments, it was confirmed that a *C. officinale* concentration of ≥150 µg/mL could dramatically decrease the physical properties of the base material, and based on this, the composition of the test group was confirmed in this study.

#### 2.2.1. Evaluation of Antimicrobial Properties

To evaluate the antimicrobial efficacy of the denture cleansers, *C. albicans* (ATCC 10231) was used. *C. albicans* was cultured in yeast mold (YM) medium (Becton Dickinson and Co., Franklin Lakes, NJ, USA) and incubated at 37 ± 1 °C for 24 h. For the inhibition zone assay, 100 µL of *C. albicans* suspension (3 × 10^5^ cells/mL) was spread on YM agar plates. Sterile paper disks (diameter, 10 mm; thickness, 1 mm) were impregnated with 20 µL of each solution and placed on the agar. After 24 h of incubation at 37 ± 1 °C, inhibition zones were measured using Vernier calipers. Each test was replicated six times, with results reported as mean values and standard deviations. The viability of *C. albicans* was assessed with the LIVE/DEAD FungaLight Yeast Viability Kit (Molecular Probes, Eugene, OR, USA) according to the manufacturer’s instructions. Equal volumes of SYTO9 dye and propidium iodide were mixed, and 3 μL of this mixture was added to 1 mL of the *C. albicans* suspension. The mixture was incubated for 15 min at room temperature in the dark, and stained samples were observed using confocal laser scanning microscopy (CLSM, LSM880; Carl Zeiss, Thornwood, NY, USA). Live cells fluoresced green, while dead cells fluoresced red. The live and dead cells were quantified using ImageJ software version 1.49 (NIH, Bethesda, MD, USA).

#### 2.2.2. Evaluation of Oral Mucosal Irritation

The oral mucosal irritation potential of the denture cleansing agents was assessed using Syrian hamsters following Part 9 of the Common Guidelines on the Biological Safety of Medical Devices (ISO 10993-10) as per the Korea Ministry of Food and Drug Safety Announcement 2014-115 [26]. Testing was conducted at the Dental Devices Testing and Evaluation Center, Yonsei University Health System. Test specimens were applied to the cheek pouches of the hamsters for at least 5 min every hour over a 4-hour period, during which microscopic examinations were performed.

### 2.3. Evaluation of Denture Cleanser on Denture Base Resin

Acrylic resin disk specimens were prepared using Jet denture resin (Lang Dental, Wheeling, IL, USA) and molded in Teflon molds with dimensions of 10 mm × 10 mm × 0.1 mm, following the manufacturer’s instructions. The Teflon mold (10.0 mm in diameter and 1.0 mm in height) was placed on a polyester film, which was then set on a microscope slide. The specimens were fully immersed in 2 mL of either control or experimental denture cleansers, placed in individual wells of a 24-well plate, and maintained at a constant temperature of 25 ± 1 °C. The *C. officinale* extract was incorporated into the denture cleansers at concentrations of 100 and 150 µg/mL, with the solutions mixed and stirred until fully dissolved. These specimens were used to observe surface properties such as hardness, roughness, and color change. The immersion process lasted for 7 and 30 days, with daily refreshing of the cleansers. After immersion, the surface of the acrylic resin disks was evaluated.

#### 2.3.1. Measurement of Microhardness

Microhardness was evaluated using a Vickers hardness tester (Microhardness Tester Dmh-2; Matuzawa Seiki, Tokyo, Japan). A 50 g load was applied for 10 s at various points on each sample, and the Vickers hardness number was recorded to assess surface hardness. Measurements were taken at two randomly selected locations per specimen, with three specimens tested per group (n = 3). Mean values and standard deviations were calculated and compared.

#### 2.3.2. Measurement of Surface Roughness

The experimental and control groups’ surface roughness was assessed using an optical surface profilometer (Contour GT; Bruker, Tucson, AZ, USA). The roughness parameter, Ra, was quantified using two-dimensional metrics. The optical profilometer captured the surface topography, displaying height variations in grayscale format. Measurements were taken at 10× magnification with a scanning area of 231 × 173 µm. The experiments were conducted in sextuplicate, and the results are presented as mean values with standard deviations.

#### 2.3.3. Measurement of Color Change

Color differences were assessed using a spectrophotometer (CM-3500d; Minolta, Kyoto, Japan). Prior to each measurement, standard calibration was performed using a white calibration plate. Specimens were evaluated based on their a*, b*, and L* values, and color differences (ΔE*) were calculated according to the CIE Lab color system. Measurements were taken at multiple locations on each specimen, with results averaged for a comprehensive assessment. The experiments were performed in sextuplicate, and the results are expressed as mean values with standard deviations.

#### 2.3.4. Measurement of Solubility

The solubility test was adapted from a previous study [14]. Disc-shaped samples (diameter, 20 mm; thickness, 1.5 mm) were placed in a desiccator for 24 h, after which their masses were recorded. Measurements of diameter (at three points) and thickness (at one central point and four peripheral points) were taken with an accuracy of 0.01 mm to calculate sample volume. Each sample was immersed in 10 mL of distilled water at 37 °C for 7 days. Subsequently, samples were rinsed, dried in a drying oven at 37 °C for 24 h until mass stabilization (with a change of <0.2 mg/h), and then weighed. Water solubility was determined by calculating the differences in mass and volume of the samples. The experiments were performed in sextuplicate, and the results are expressed as mean values with standard deviations.

#### 2.3.5. Evaluation of Biological Properties

The cytotoxicity of the test materials was assessed using diffusion through the agar method, following ISO 10993-5:2009 and ISO 7405:2018 standards [27]. Cell suspensions (2.5 × 10^5^ cells/mL) in 10 mL volumes were seeded into 100 mm diameter cell culture dishes (SPL, Pocheon-Si, Republic of Korea) and incubated at 37 °C in a humidified environment with 5% carbon dioxide. After 24 h, the medium was replaced with 10 mL of freshly prepared agar medium containing 2 × RPMI 1640 (Sigma, Irvine, Ayrshire, UK). Once the liquid culture medium solidified, 10 mL of neutral red solution (0.01% in phosphate-buffered saline; Sigma, St. Louis, MO, USA) was added in the dark for 20 min. The excess neutral red solution was removed, and test samples were placed on the agar surface alongside positive controls (latex sheet) and negative controls (Teflon mold) in the same cell culture dish. After a 24-hour incubation period, decolorization and lysis indices were evaluated using an optical microscope according to ISO 7405 standards. Decolorized zones were rated as follows: 0 = no detectable decolorization; 1 = decolorization only under the specimen; 2 = decolorization within a zone ≤ 5 mm from the specimen; 3 = decolorization within a zone ≤ 10 mm from the specimen; 4 = decolorization within a zone >10 mm from the specimen; and 5 = total culture decolorization. Cell lysis, defined as the loss of cell membrane integrity observed under light microscopy, was scored as follows: 0 = no detectable cell lysis; 1 = < 20% cell lysis; 2 = 20–40% cell lysis; 3 = 40–60% cell lysis; 4 = 60–80% cell lysis; and 5 = > 80% cell lysis. The experiments were performed in sextuplicate.

#### 2.3.6. Measurement of Polyphenol and Flavonoid Contents

To determine polyphenol and flavonoid contents, *C. officinale* extract powder was dissolved in distilled water to prepare solutions of 100 and 150 µg/mL. The solutions were stored at 37 ± 1 °C in a water bath for 24 h before analysis. For the polyphenol assay, 50 µL of the extract solution was mixed with 650 µL of distilled water, followed by the addition of 50 µL of Folin–Denis reagent. After a 3 min reaction, 100 µL of 10% sodium carbonate solution and 150 µL of distilled water were added to achieve a final volume of 1 mL. After incubation for 1 h, the absorbance was measured at 725 nm using a visible-ultraviolet spectrophotometer (X-ma 1200 Spectrophotometer, Human Corp., Seoul, Republic of Korea). A standard curve was established using gallic acid (Sigma-Aldrich, St Louis, MO, USA) at concentrations of 20, 40, 60, 80, and 100 µg/mL. For the flavonoid content analysis, 1 mL of diethylene glycol was added to 100 µL of the extract solution, followed by 100 µL of 1 N sodium hydroxide. The mixture was incubated at 37 °C for 1 h, and the absorbance was measured at 420 nm using a visible-ultraviolet spectrophotometer. A standard curve was created using naringin (Sigma-Aldrich, St Louis, MO, USA) at concentrations of 20, 40, 60, 80, and 100 µg/mL to quantify the flavonoid content. Polyphenols and flavonoids were measured in six independent experiments.

### 2.4. Statistical Analysis

All statistical analyses were performed using SPSS 23 software (IBM Corp., Armonk, NY, USA). Differences among groups were analyzed using one-way analysis of variance, followed by Tukey’s post-hoc test, with a significance level set at 0.05.

## 3. Results

### 3.1. Antimicrobial and Biological Properties

#### 3.1.1. Evaluation of Antimicrobial Properties

**Figure 1:**
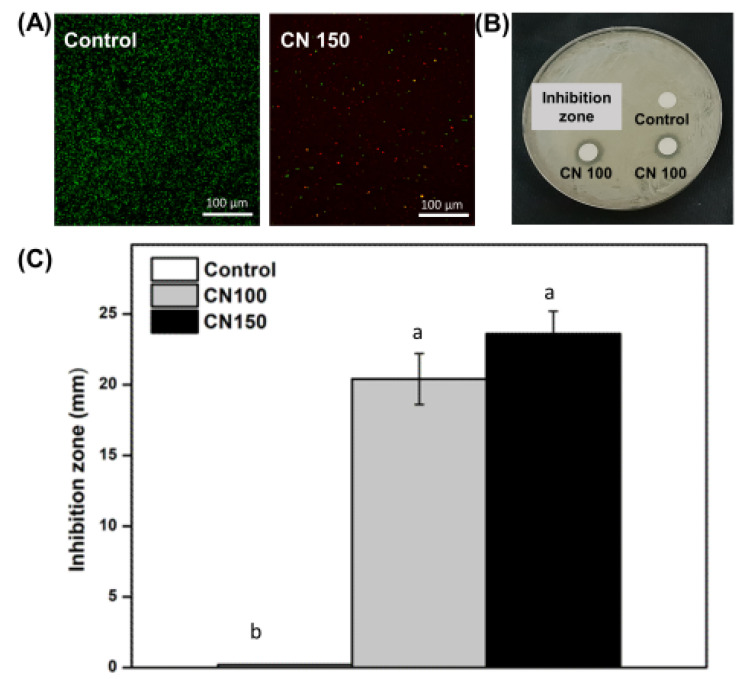
Antifungal properties: (**A**) Representative live/dead staining images of *Candida albicans*, (**B**) representative agar plate showing inhibition zone, (**C**) diameter of inhibition zones on agar plates. Different lowercase letters above the bars indicate significant differences. CN 100, the group with 100 µg/mL of *C. officinale*; CN 150, the group with 100 µg/mL of *C. officinale*.

The results of the fungal viability staining are depicted in Figure 1A. The control group exhibited more fungi stained with green fluorescence, indicating greater viability. In contrast, the CN150 group displayed fewer live fungi and a higher proportion of dead fungi stained red. Antifungal activity against *C. albicans* was assessed using the inhibition zone assay, as shown in Figure 1B,C. After 24 h of incubation, antifungal activity increased with the addition of *C. officinale* extract. The inhibition zones of the CN 100 and CN 150 groups were significantly larger compared to the control group (*p* < 0.05).

#### 3.1.2. Evaluation of Oral Mucosal Irritation

**Figure 2:**
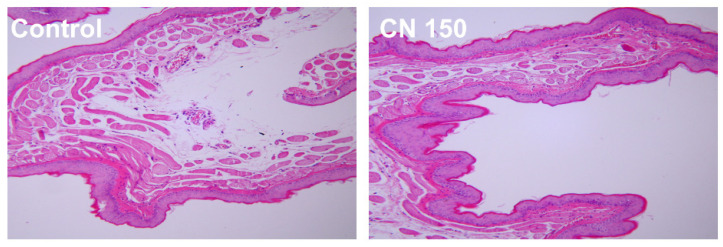
Representative oral mucosal irritation images at 20 × magnification.

Oral mucosal irritation was assessed, with representative images shown in Figure 2. No abnormalities were observed in the experimental animals throughout the observation period. Histological examination of cheek pouches 24 h post-contact revealed a numeric grade difference of 0.3 between the test and control groups, indicating minimal irritation. 

### 3.2. Evaluation of Denture Cleanser Effects

#### 3.2.1. Measurement of Microhardness

**Figure 3:**
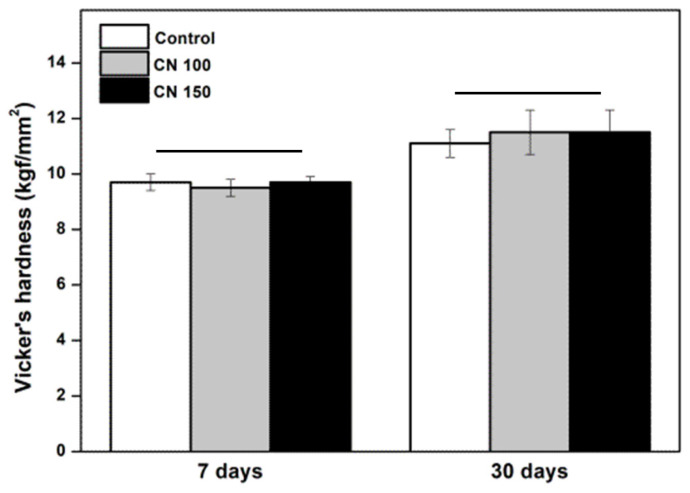
Comparison of microhardness. There are no significant differences between the groups indicated by the horizontal bars (*p* > 0.05).

The surface hardness of the denture base resins treated with the denture cleaning agents was evaluated after 7 and 30 days of storage. No significant changes in microhardness were observed between the control and experimental groups (CN 100 and CN 150) (*p* > 0.05) (Figure 3).

#### 3.2.2. Measurement of Surface Roughness

**Figure 4:**
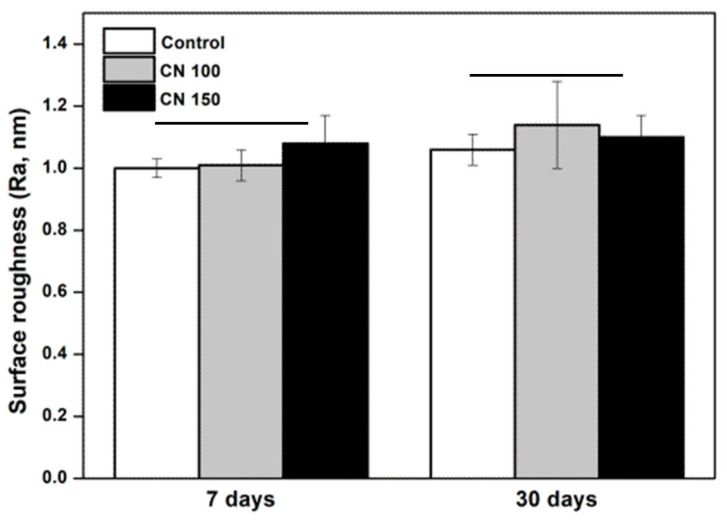
Comparison of surface roughness. There are no significant differences between the groups indicated by the horizontal bars (*p* > 0.05).

The surface roughness of the denture base resins treated with the denture cleaning agents was measured after 7 and 30 days of storage. No significant changes in surface roughness were noted between the control and experimental groups (CN 100 and CN 150) (*p* > 0.05) (Figure 4).

#### 3.2.3. Measurement of Solubility

**Figure 5:**
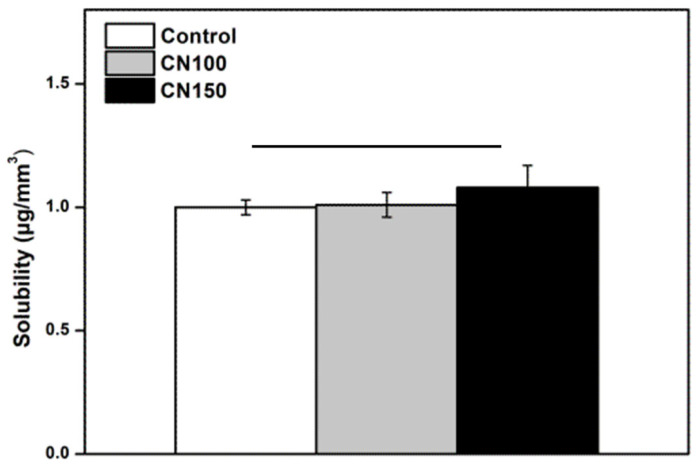
Comparison of solubility. There are no significant differences between the groups indicated by the horizontal bars (*p* > 0.05).

The solubility of the denture cleaning agents containing natural extracts showed no significant difference in solubility between the experimental groups (CN 100 and CN 150) and the control group (*p* > 0.05) (Figure 5).

#### 3.2.4. Measurement of Color Change

**Figure 6:**
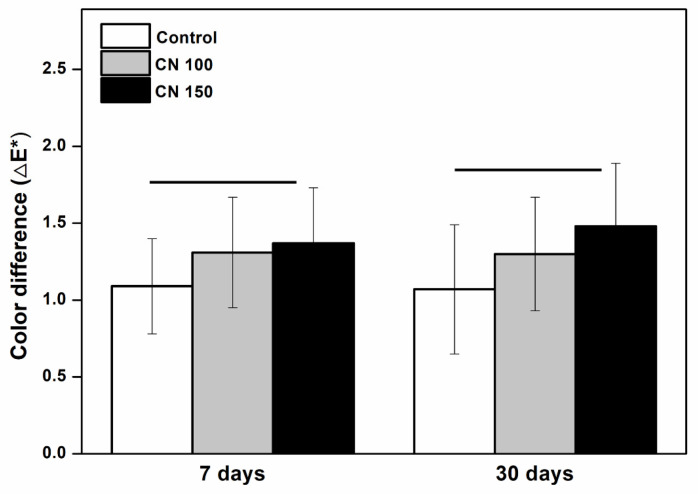
Comparison of color difference. There are no significant differences between the groups indicated by the horizontal bars (*p* > 0.05).

The color stability of the denture base resin was evaluated by measuring the total color change (△E*) over 7 and 30 days. The △E* values for the experimental groups (CN 100 and CN 150) did not differ significantly from the control group after both time points (*p* > 0.05), indicating no significant color change (Figure 6).

#### 3.2.5. Evaluation of Biological Properties

**Figure 7:**
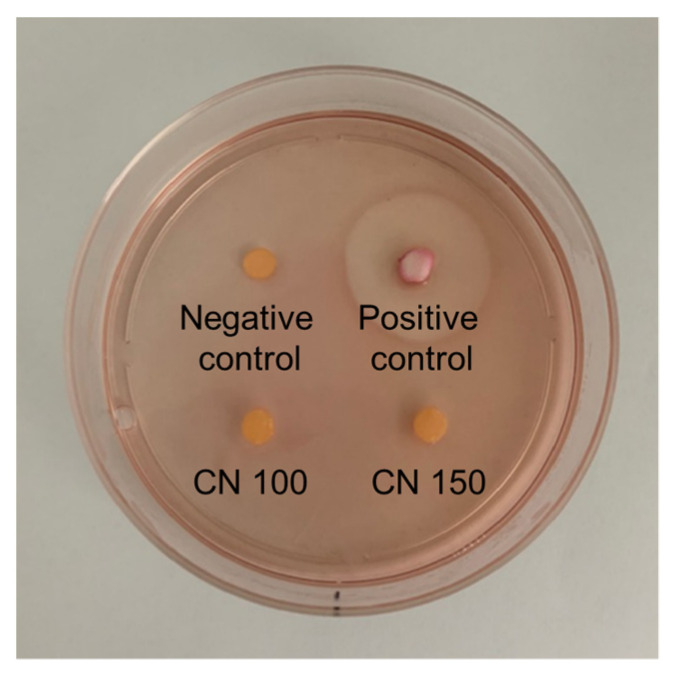
Representative image from the agar diffusion test.

The cytotoxicity of the denture cleaning agents was assessed using the agar overlay test. The decolorization and lysis indices are presented in Table 1 and Figure 7. No abnormalities or decolorization were observed in the cells beneath or around the specimens containing natural extracts. Cytotoxicity assessment indicated that none of the experimental groups exhibited significant cytotoxicity, as indicated by the response indices compared to the negative and positive controls.

#### 3.2.6. Evaluation of Phenolic and Flavonoid Contents

Quantification of polyphenols and flavonoids in the *C. officinale* extract revealed the following: the CN 100 group had a polyphenol content of 8.3 ± 2.1 µg/mL and a flavonoid content of 17.1 ± 0.9 µg/mL. The CN 150 group showed a polyphenol content of 9.8 ± 2.5 µg/mL and a flavonoid content of 17.4 ± 1.4 µg/mL. Increasing the concentration of *C. officinale* extract resulted in a statistically significant increase in the polyphenol and flavonoid contents (*p* < 0.05) (Table 2).

## 4. Discussion

Acrylic resins are widely used in prosthodontics, with significant advancements over time improving their physical properties and biocompatibility [3]. These materials are primarily used for fabricating complete and partial dentures [28]. For patients with partial or complete edentulism using removable prostheses, maintaining proper hygiene of the remaining dentition, gingiva, and dentures is essential for prolonging the lifespan of the prosthesis [1]. Consequently, specialized oral hygiene products or cleansers are recommended [21]. However, most commercially available denture cleansers contain synthetic chemical ingredients, which have been reported to alter the surface characteristics of denture base resins and may induce cytotoxic effects upon contact with oral tissues [8]. In this study, 1% cocamidopropyl betaine solution was chosen as the surfactant for the denture cleanser formulations. Cocamidopropyl betaine is widely used in personal care products such as shampoos, liquid soaps, and cleansers owing to its mildness and low irritation potential [29]. Its gentle nature makes it suitable for sensitive skin and oral applications [21,29]. Additionally, it enhances the performance of other surfactants while minimizing irritation to skin and mucous membranes, making it an ideal choice for ensuring the safety and efficacy of the denture cleanser [29]. This study aimed to develop a novel denture cleanser containing natural plant extracts from *C. officinale* and evaluate its antimicrobial efficacy, as well as its impact on the physicochemical properties, color stability, and biocompatibility of denture base resin.

Antifungal experiments revealed that the denture cleanser containing *C. officinale* exhibited superior antifungal activity compared to the control group (Figure 1). *C. officinale*, traditionally used as a medicinal plant, has been increasingly recognized for its antimicrobial properties [19]. In this study, the higher concentration group, CN 150, was selected to assess live/dead cell viability. Based on the inhibition zone test results, it was anticipated that the CN 100 group would exhibit a similar trend. Studies have shown that extracts of *C. officinale* are effective against various bacterial pathogens, including *Staphylococcus aureus*, *Pseudomonas aeruginosa*, and *Escherichia coli* [24,25]. Additionally, extracts of *C. officinale* have shown activity against methicillin-resistant *Staphylococcus aureus* [24]. The antimicrobial action of *C. officinale* is attributed to various mechanisms, with polyphenols and flavonoids identified as key active components [30]. These compounds inhibit bacterial cell wall synthesis, disrupt cell membranes, compromise cellular structural integrity, and induce oxidative stress, leading to bacterial damage and impaired survival [20]. Additionally, they have been shown to exert direct inhibitory effects on bacteria [31]. Thus, the polyphenols and flavonoids in the *C. officinale* extract contribute significantly to its observed antimicrobial effects (Table 2). In this study, we focused on measuring the polyphenol and flavonoid content in *C. officinale* extract owing to their known antioxidant and antimicrobial properties. Phenolic compounds, including polyphenols and flavonoids, are recognized for their ability to disrupt microbial processes, such as cell wall synthesis and membrane integrity, impacting microbial survival. Their antibacterial activity and antioxidant effects have been well-documented [20,23,25]. Our analysis revealed significant levels of polyphenols and flavonoids in the *C. officinale* extract, suggesting that the observed antimicrobial efficacy of our denture cleanser formulations is attributed to these bioactive substances. Specifically, polyphenols and flavonoids directly inhibit microbial proliferation and enhance antimicrobial activity [24]. These findings indicate that incorporating *C. officinale* extract into denture cleansers not only boosts their antibacterial efficacy but also leverages the beneficial effects of phenolic compounds observed in similar studies [20,31].

Denture cleaning methods and cleansers should not adversely affect the properties of denture base resin [1,14]. Previous research has reported that denture cleansers can impact the mechanical strength, color stability, and surface morphology of denture base resins [4]. Improper mechanical cleaning can cause resin wear, leading to abrasion-induced scratches that increase surface roughness and promote plaque and calculus accumulation [5]. Therefore, evaluating the hardness of the resin following exposure to denture cleansers is essential [14]. Surface hardness is indicative of the resin’s resistance to masticatory forces, and a reduction could result in uneven stress distribution during mastication [5,23]. Increased surface roughness can also lead to greater microbial adherence, staining, plaque accumulation, food residue build-up, and unpleasant odors [10]. The results of the present study showed that the denture cleanser containing *C. officinale* did not adversely affect the hardness or roughness of the denture base resin (Figure 3 and Figure 4). The observed increase in microhardness after 30 days is likely due to the natural hardening process of polymethyl methacrylate, where the material’s surface layer becomes more solid over time [1]. 

Traditional denture cleansers often contain alkaline hypochlorites, which are effective in stain removal and dissolving mucin and other organic materials [7,15]. However, these substances can cause ocular and dermal irritation, corrode metals, and bleach denture base resins [21,32]. Chlorhexidine gluconate is commonly used to manage denture stomatitis caused by *Candida* species, but its daily use is often limited owing to its tendency to stain dentures [6,8]. In contrast, the *C. officinale* extract used in this study did not lead to any discoloration of the denture base resin (Figure 5). To ensure precise detection of color changes, we used a spectrophotometer rather than relying solely on visual inspection. The results showed no significant color differences between the control and experimental groups, and no detectable color changes were observed to the naked eye. Therefore, the inclusion of *C. officinale* extract did not negatively affect the color stability of the acrylic resin.

Similarly, it is essential that denture base resin maintains its stability and does not dissolve in the oral cavity, as this would compromise its structural integrity [1,14]. The solubility test results revealed no significant differences between the experimental and control groups (Figure 6).

As denture cleansers are in direct contact with oral mucosa, assessing their cytotoxicity is crucial [32]. Previous studies using the MTT assay confirmed that *C. officinale* does not exhibit cytotoxicity [20,23]. Since denture cleansers come into direct contact with mucosal surfaces, we evaluated cytotoxicity using the agar diffusion test in this study. The agar overlay test, used to assess cytotoxicity [27,33], involves the direct application of materials on a solid medium, allowing for visual confirmation of the affected cell range [27]. Our results showed that *C. officinale* extract did not exhibit cytotoxicity at any tested concentration (Table 1). Additionally, oral mucosal irritation was assessed using hamster models, a common practice for evaluating the potential irritation of dental products [34,35]. Daily clinical observations (irritation scores calculated based on previous studies) [16,34] and histological examinations of mucosal damage revealed no significant abnormalities, indicating that the denture cleanser containing *C. officinale* has minimal potential for mucosal irritation and is considered safe for clinical use (Figure 2). We designated the high-concentration experimental group as a representative for animal testing to minimize unnecessary animal sacrifice, focusing on the higher concentration to assess potential effects more efficiently.

The experimental groups utilized *C. officinale* at concentrations of 100 and 150 μg/mL, combined with 1% natural surfactant, cocamidopropyl betaine. Previous studies have demonstrated the antimicrobial properties of *C. officinale* [20,23], with Lee et al. (2020) [20] reporting significant efficacy at concentrations ranging from 100 to 200 µg/mL against various pathogens, including *C. albicans.* However, concentrations of 200 µg/mL showed cytotoxicity, raising safety concerns. Therefore, 100 and 150 µg/mL were chosen for this study to balance efficacy and safety, ensuring effective antimicrobial activity while minimizing potential cytotoxic effects.

The first null hypothesis, which stated that varying concentrations of *C. officinale* extract in denture cleansers would not significantly alter their antimicrobial and biocompatibility properties, was confirmed. Additionally, no significant changes in the surface properties or dissolution of the denture base resin were observed with the experimental denture cleanser, irrespective of the concentration of *C. officinale* extract. Consequently, the second null hypothesis—that the inclusion of *C. officinale* extract would not significantly affect the surface properties or dissolution of the denture material—was also upheld.

Although the newly developed denture cleanser containing *C. officinale* extract maintained the surface, physical, and biological properties of the acrylic resin without adverse effects, further long-term studies are necessary to confirm the safety and efficacy of denture base resins. Additionally, it is crucial to consider the interaction between the denture cleanser and the bond strength of denture liners, as this is a critical factor for patients using dentures [5]. Future research should focus on evaluating the impact of prolonged exposure to denture cleansers on the bond strength between denture base resins and liners to establish their clinical relevance.

## 5. Conclusions

This study evaluated the antifungal activity of a novel denture cleanser containing *C. officinale* extract and its impacts on the surface characteristics, physical properties, and cytotoxicity of denture base resins. In vitro assessments revealed no significant differences in surface hardness, roughness, solubility, color change, or biocompatibility between the experimental and control groups (*p* > 0.05). Notably, the denture cleanser containing *C. officinale* extract exhibited significant antifungal efficacy against *C. albicans* (*p* < 0.05). These results highlight *C. officinale* extract as a promising antimicrobial agent for denture hygiene. Importantly, the incorporation of *C. officinale* in the denture cleanser demonstrated that it does not adversely affect the physical, mechanical, or cytotoxic characteristics of the denture base material. This suggests that *C. officinale* could be a valuable component in the development of natural denture cleansers, offering a safer alternative to synthetic chemicals. Integrating *C. officinale* into denture cleansers will not only preserve the physical integrity of denture materials but also enhance antimicrobial protection, potentially reducing adverse effects on oral tissues and promoting overall oral health. Future research should focus on optimizing the formulation and evaluating its long-term clinical efficacy to maximize the benefits of *C. officinale* extracts in preventing oral stomatitis and enhancing overall denture hygiene.

## Figures and Tables

**Table 1:** Antifungal activity results of the agar diffusion test.

Test Materials	Decolorization (cm)	Decolorization Index	Lysis Index	Interpretation
Positive control	2.6 ± 0.15	4	5	Severely cytotoxic
Negative control	0	0	0	Non-cytotoxic
CN 100	0	0	0	Non-cytotoxic
CN 150	0	0	0	Non-cytotoxic

CN 100, the group with 100 µg/mL of *C. officinale*; CN 150, the group with 100 µg/mL of *C. officinale*.

**Table 2:** Quantification of polyphenol and flavonoid content in *Cnidium officinale* extracts.

Experimental Group	Polyphenol Content (μg/mL)	Flavonoid Content (μg/mL)
CN 100	8.3 ± 2.1 ^A^	17.1 ± 0.9 ^B^
CN 150	9.8 ± 2.5 ^A^	17.4 ± 1.4 ^B^

Different superscript letters denote significant differences between experimental groups for each component (*p* < 0.05).

## Data Availability

The original contributions presented in the study are included in the article; further inquiries can be directed to the corresponding authors.
